# Attention-Fused Staged DWT-LSTM for Fault Diagnosis of Embedded Sensors in Asphalt Pavement

**DOI:** 10.3390/ma18163917

**Published:** 2025-08-21

**Authors:** Jiarui Zhang, Haihui Duan, Songtao Lv, Dongdong Ge, Chaoyue Rao

**Affiliations:** 1 National Engineering Research Center of Highway Maintenance Technology, Changsha University of Science & Technology, Changsha 410114, China; blzjr@stu.csust.edu.cn (J.Z.); lst@csust.edu.cn (S.L.); 18390079778@stu.csust.edu.cn (C.R.); 2 National Key Laboratory of Green and Long-Life Road Engineering in Extreme Environment (Changsha), Changsha University of Science & Technology, Changsha 410114, China

**Keywords:** asphalt pavement, state monitoring, sensor, fault diagnosis, DWT-LSTM, attention mechanism

## Abstract

Fault diagnosis for embedded sensors in asphalt pavement faces significant challenges, including the scarcity of real-world fault data and the difficulty in identifying compound faults, which severely compromises the reliability of monitoring data. To address these issues, this study proposes an intelligent diagnostic framework that integrates a Discrete Wavelet Transform (DWT) with a staged, attention-based Long Short-Term Memory (LSTM) network. First, various fault modes were systematically defined, including short-term (i.e., bias, gain, and detachment), long-term (i.e., drift), and their compound forms. A fine-grained fault injection and labeling strategy was then developed to generate a comprehensive dataset. Second, a novel diagnostic model was designed based on a “Decomposition-Focus-Fusion” architecture. In this architecture, the DWT is employed to extract multi-scale features, and independent sub-models—a Bidirectional LSTM (Bi-LSTM) and a stacked LSTM—are subsequently utilized to specialize in learning short-term and long-term fault characteristics, respectively. Finally, an attention network intelligently weights and fuses the outputs from these sub-models to achieve precise classification of eight distinct sensor operational states. Validated through rigorous 5-fold cross-validation, experimental results demonstrate that the proposed framework achieves a mean diagnostic accuracy of 98.89% (±0.0040) on the comprehensive test set, significantly outperforming baseline models such as SVM, KNN, and a unified LSTM. A comprehensive ablation study confirmed that each component of the “Decomposition-Focus-Fusion” architecture—DWT features, staged training, and the attention mechanism—makes an indispensable contribution to the model’s superior performance. The model successfully distinguishes between “drift” and “normal” states—which severely confuse the baseline models—and accurately identifies various complex compound faults. Furthermore, simulated online diagnostic tests confirmed the framework’s rapid response capability to dynamic faults and its computational efficiency, meeting the demands of real-time monitoring. This study offers a precise and robust solution for the fault diagnosis of embedded sensors in asphalt pavement.

## 1. Introduction

The intelligent operation and maintenance of modern road infrastructure are highly dependent on long-term, high-fidelity in situ monitoring data. Sensors embedded within asphalt pavements serve as a critical means of acquiring internal structural responses and evaluating service status [1,2]. However, subjected to harsh service conditions—including construction disturbances, high-frequency cyclic loading, steep temperature gradients, and environmental corrosion—these sensors are prone to performance degradation and various types of faults. This leads to distorted monitoring data, which can subsequently trigger erroneous maintenance decisions. Therefore, developing a precise, efficient, and robust automated fault diagnosis method for these sensors is of great significance for ensuring data quality and solidifying the foundation for concepts such as “smart roads”.

As a core component for ensuring the reliability of Structural Health Monitoring (SHM) systems, sensor fault diagnosis has evolved along several technological paths. Model-based diagnostic methods identify faults by comparing the residuals between predictions from a mathematical or physical model and actual measurements [3]. Yan et al. [4] applied the generalized quasi-natural ratio test for self-diagnosis of sensors in building SHM systems, while Lydakis et al. [5] identified sensor faults by establishing an overdetermined system between measurement signals and the actual structural motion. Li et al. [6] utilized the generalized likelihood ratio and correlation coefficients to detect sensor faults in bridge SHM systems. Although such methods are physically interpretable, their diagnostic accuracy is highly dependent on model fidelity. Establishing a high-fidelity model for a system as complex as an asphalt pavement—characterized by material nonlinearity [7,8,9], intricate interlayer interactions, and variable loading and environmental conditions—is exceptionally challenging.

To overcome this limitation, data-driven fault diagnosis methods have emerged. Their primary advantage lies in their ability to learn fault patterns directly from monitoring data, thereby circumventing the need for precise models and demonstrating greater flexibility and applicability in complex systems. Traditional data-driven approaches rely on signal processing techniques for feature extraction, such as Principal Component Analysis (PCA) [10,11] and Wavelet Transform (WT) [12]. However, PCA’s inherent linear assumptions hinder its ability to capture nonlinear relationships [13,14], while the performance of WT is sensitive to the choice of wavelet basis and decomposition levels [15,16], and its robustness and sensitivity may be limited in the context of strong background noise and weak fault signals in road engineering.

In recent years, Artificial Intelligence (AI) techniques, particularly machine learning and deep learning, have gained prominence in fault diagnosis due to their powerful nonlinear mapping and automatic feature learning capabilities [17,18,19]. While traditional machine learning methods like Support Vector Machine (SVM) [20] and K-Nearest Neighbors (KNN) [21] have been applied, their effectiveness is constrained by the quality of hand-crafted features. Deep learning methods, on the other hand, show immense potential by automatically learning deep, abstract features from raw or minimally processed data [22]. Jana et al. [23] combined a CNN and a CAE for real-time fault handling; Tang et al. [24] utilized a CNN to fuse multi-source information for sensor anomaly detection; and Li et al. [25] employed a Transformer-enhanced DenseNet to achieve precise fault localization. Furthermore, promising results have been achieved using LSTM-based fault classification and isolation [26,27], specific deep learning architectures for fault detection and signal reconstruction [28,29,30], multi-instance learning for sensor failure problems [31], and hybrid methods combining wavelet analysis with neural networks [32,33]. Notably, the Attention Mechanism, a state-of-the-art technique, has already been shown to significantly enhance diagnostic accuracy in complex engineering systems by enabling models to dynamically focus on critical segments of time-series data [34,35,36].

However, a significant disconnect persists between these advanced techniques and their application within pavement engineering. The application of deep learning is a relatively recent development in pavement engineering, a field historically dominated by research into materials and structures [37,38,39]. Current deep learning research in the field is predominantly focused on “downstream” tasks, such as pavement performance prediction [40,41,42] and image-based distress detection [43,44,45], which implicitly assume the integrity and accuracy of their underlying data sources. This prevailing oversight highlights a critical research gap: the absence of robust diagnostic methods for the “upstream,” foundational challenge of ensuring embedded sensor reliability. This gap is not accidental; it stems from two fundamental and intertwined challenges inherent to the domain: (1) Scarcity of Domain-Specific Fault Knowledge and Data: The operational environment for sensors embedded in asphalt—subjected to extreme temperatures, compaction pressures, and being non-replaceable—is unique. Consequently, their failure modes, evolutionary paths, and signal signatures remain poorly characterized. Moreover, the difficulty in acquiring well-labeled, in-service fault data presents a formidable barrier to developing and validating specialized data-driven models. (2) Inherent Complexity of Compound Faults: Under real-world conditions, sensor failures rarely occur in isolation. Instead, they often manifest as “compound faults,” where multiple fault mechanisms coexist, producing complex and latent signal patterns. These composite failures pose a severe challenge to the robustness and resolution of existing methods, which are ill-equipped for such diagnostic complexity.

To address these challenges, this paper introduces a problem-driven, intelligent diagnostic framework that makes three primary contributions: (1) Systematic Problem Formulation. This study systematically defined the unique failure modes of embedded pavement sensors, including compound fault. It further proposed a novel sample construction method to resolve the representational challenges posed by heterogeneous time-scale features in the sensor data. (2) A Novel “Decompose-Focus-Fuse” Architecture. A specialized architecture was designed to tackle feature heterogeneity, employing parallel, independently pre-trained sub-models to “focus” on two disparate feature sets: short-term statistical features and long-term wavelet coefficients. (3) Attention-Based Intelligent Fusion. An attention mechanism is leveraged to intelligently weigh and fuse the outputs of the expert sub-models. This targeted fusion is critical for accurately decoupling and identifying complex compound-mode failures that are intractable for conventional methods. This model employs a “Decomposition-Focus-Fusion” strategy to efficiently and accurately identify eight sensor operational states, including complex compound faults, thereby providing high-quality foundational data to support the perception, interpretation, diagnosis, and evaluation of pavement performance.

## 2. Fault Mode Definition and Sample Construction for Embedded Pavement Sensors

Effective fault diagnosis for sensors relies on a comprehensive and realistic fault dataset. In the context of asphalt pavement engineering, however, obtaining labeled real-world fault data is exceptionally difficult due to two primary challenges: data scarcity and labeling complexity. To address this, this study employs a fine-grained fault injection and labeling strategy to generate a synthetic dataset. This strategy systematically defines typical sensor fault modes and resolves the challenge of integrating fault features across different time scales during sample construction.

### 2.1. Fault Mode Definition

Based on their temporal characteristics, sensor faults are categorized into three types: Short-Term Faults, characterized by sudden, second-scale changes and anomalous fluctuations; Long-Term Faults, characterized by slow, continuous baseline deviations over minutes, hours, or longer; and Compound Faults, where both types occur concurrently.

#### 2.1.1. Short-Term Faults

Short-term faults encompass a range of modes, including complete failure (e.g., no signal), high-frequency noise, freezing (i.e., a stuck constant value), and calibration errors. Calibration errors typically manifest in the signal as bias or gain faults, which are already core components of this study. While faults like complete failure or freezing are important, they often produce highly distinct and readily identifiable signatures (e.g., zero or constant variance) that can be effectively detected by simpler statistical checks. Therefore, this study deliberately focuses on three classic and more challenging short-term faults whose signal characteristics are subtle and easily confused with normal operation or each other and thus pose a greater diagnostic challenge: Bias, Gain, and Detachment.

(1) Bias

**Figure 1:**
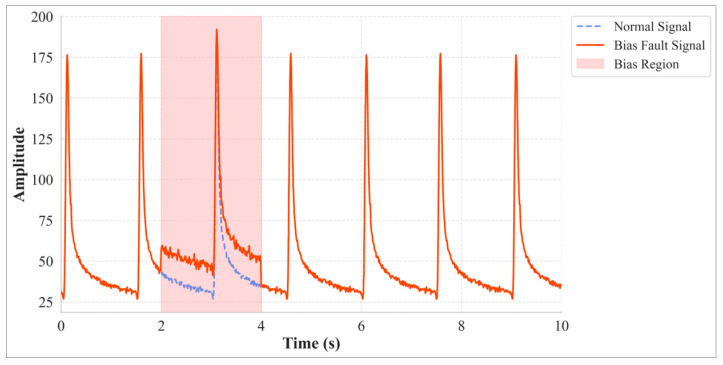
Bias Fault Example.

A bias fault manifests as a nearly constant offset superimposed on the sensor’s output signal, often accompanied by some level of noise fluctuation (Figure 1). For embedded pavement sensors, this can be caused by aging of a sensing element, deformation of a packaging material, or micro-changes in the interface state between the sensor and pavement structure. Its mathematical model can be expressed as:

(1) $$y_{0}\left(t\right)=\;y\left(t\right)+\;w\;+\;\sigma_{b}\left(t\right)$$

where $y\left(t\right)$ is the original clean signal, $y_{0}\left(t\right)$ is the faulty signal (as in subsequent equations), w is the bias magnitude, and $\sigma_{b}\left(t\right)$ is additive Gaussian white noise.

(2) Gain

**Figure 2:**
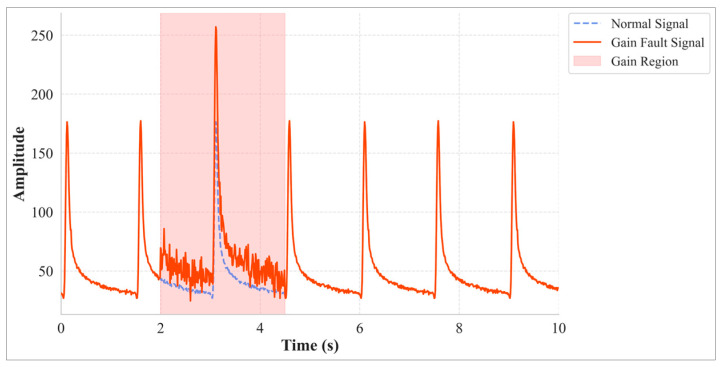
Gain Fault Example.

A gain fault refers to an unintended change in sensor sensitivity, leading to a systematic amplification of the output signal’s amplitude (Figure 2). This typically originates from alterations in the sensing element’s response characteristics or a failure of calibration parameters. Its model is represented as:

(2) $$y_{0}\left(t\right)=\;k\cdot y\left(t\right)+\;\sigma_{g}\left(t\right)$$

where k is the gain factor simulates the abnormal signal amplification, and *σ_g_*(*t*) is additive Gaussian white noise.

(3) Detachment

**Figure 3:**
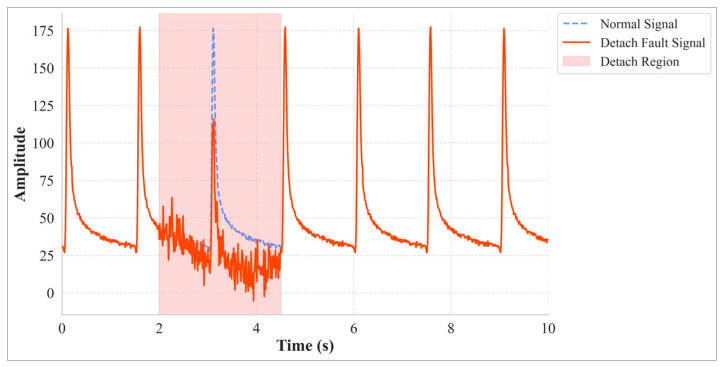
Detachment Fault Example.

A detachment or decoupling fault occurs when the mechanical transfer path between the sensor and the host pavement structure deteriorates or is interrupted. This prevents the sensor from accurately perceiving the true changes in the structural response, manifesting as a significant signal amplitude decay accompanied by abnormal drift and increased noise (Figure 3). Common causes include adhesive aging, interface delamination, or improper installation. Its model combines signal decay, a drift term, and a noise term:

(3) $$y_{0}\left(t\right)=\;y\left(t\right)\cdot e^{-\alpha \cdot f\left(t^{\prime }\right)}\;+\;d\left(t^{\prime }\right)+\;\sigma_{d}\left(t^{\prime }\right)$$

where *t*′ is the time elapsed since the detachment fault began, $e^{-\alpha \cdot f\left(t^{\prime }\right)}$ is an exponential decay factor representing the signal attenuation, and the function of time *f*(*t*′) is implemented as a power-law function of normalized time. *d*(*t*′) represents a potential free oscillation or irregular drift component after decoupling, and *σ_d_*(*t*′) is additive random noise.

#### 2.1.2. Long-Term Fault

**Figure 4:**
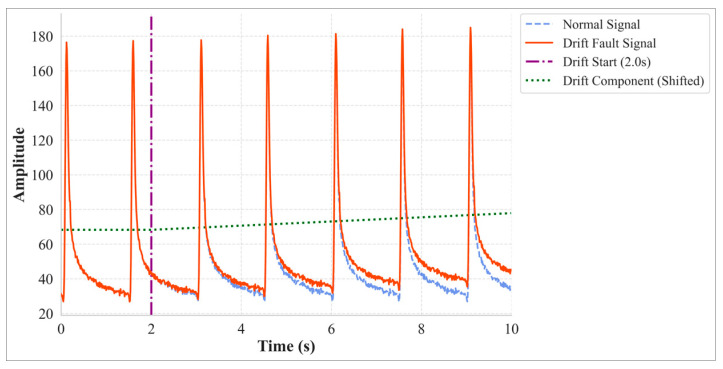
Drift Fault Example.

Drift is the archetypal long-term fault, characterized by a slow, continuous, and systematic deviation of the sensor’s baseline over minute-to-hour time scales (Figure 4). It is typically caused by factors such as sensor element aging, packaging material creep, and the cumulative effects of uncompensated long-term temperature variations. This study employs a combined linear and quadratic model to simulate this process:

(4) $$y_{0}\left(t\right)=\;y\left(t\right)+\;d\left(t\right)$$

where the drift term *d*(*t*) begins to increase at *t* > *t_start_* and is defined as:

(5) $$d\left(t\right)=\;K_{1}\cdot \left(t\;-\;t_{start}\right)+K_{2}\cdot {\left(t\;-\;t_{start}\right)}^{2}$$

$K_{1}\cdot \left(t\;-\;t_{start}\right)$ represents a quasi-linear drift trend, often caused by factors like periodic temperature changes, while $K_{2}\cdot {\left(t\;-\;t_{start}\right)}^{2}$ represents a nonlinear trend, often associated with accelerated material aging. The drift rate coefficients *K*_1_ and *K*_2_ are determined based on the signal’s dynamic range and the expected severity of the drift.

#### 2.1.3. Compound Faults

**Figure 5:**
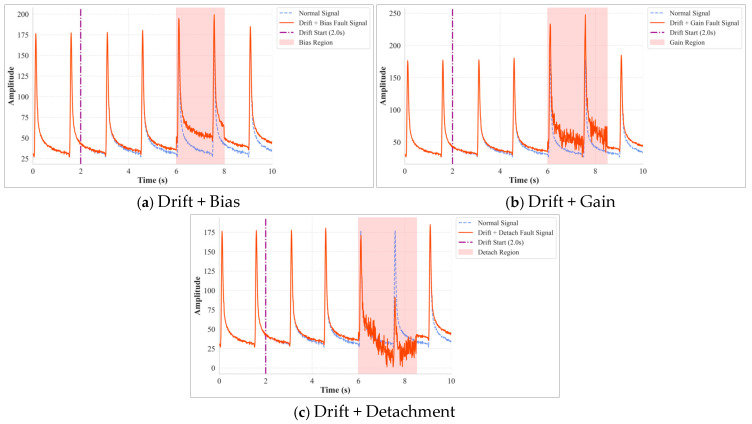
Compound Fault Example.

Under complex in-service conditions, an embedded pavement sensor may experience the concurrent or sequential effects of multiple fault modes, forming a Compound Fault. In this study, a Compound Fault specifically refers to the co-occurrence of a long-term fault (drift) and a short-term fault (bias, gain, or detachment). As shown in Figure 5, the features of such faults are complex and subtle, differing only slightly from their single-fault counterparts and thus posing a greater challenge to diagnostic models.

To ensure the diversity and realism of the generated samples, the key parameters in the fault models described in this section were randomly drawn from predefined ranges that are both physically meaningful and produce distinct fault features. The detailed parameters and their value ranges are provided in Table 1.

### 2.2. Differentiated Sample Construction Method

**Figure 6:**
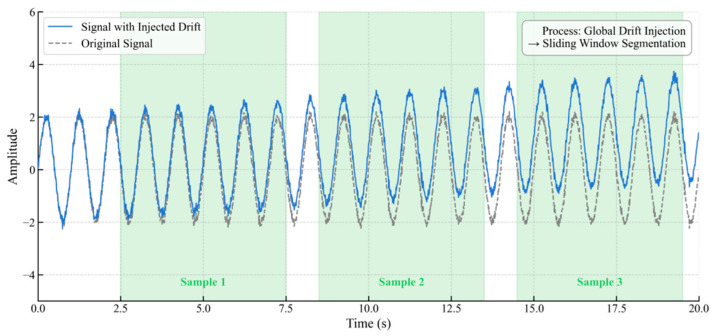
Drift Fault Sample Construction.

**Figure 7:**
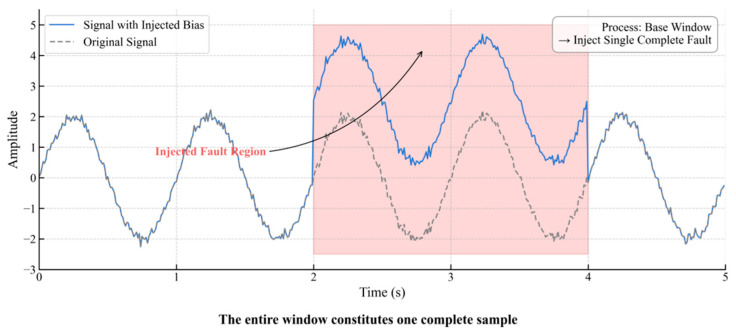
Short-Term Fault Sample Construction.

**Figure 8:**
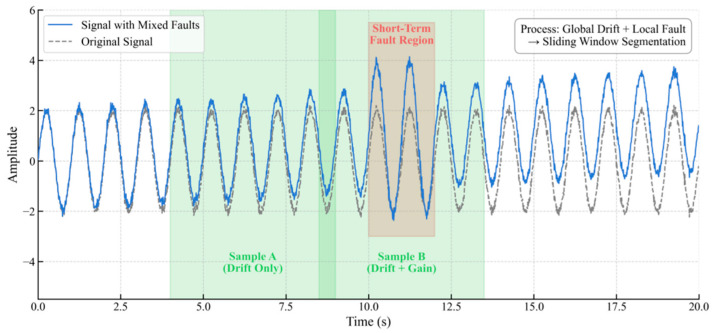
Compound Fault Sample Construction.

To address the significant feature disparities between short-term (e.g., bias) and long-term (e.g., drift) faults and their conflicting requirements for analysis window length, this study proposes a differentiated sample construction strategy. The core of this strategy is to establish a “baseline analysis window length” sufficient to capture the complete evolution of a long-term fault and then apply customized injection and labeling rules for different fault types within this unified dimension. This approach aims to maximize the distinctiveness of each fault’s features while maintaining a consistent input dimension for the model, thereby providing optimized data for subsequent staged training. The construction logic is as follows:

(1) Drift Fault Sample Construction: As shown in Figure 6, a drift fault is injected globally into a long base signal, which is then segmented using a sliding window of the “baseline analysis window length.” To effectively distinguish true drift from normal baseline fluctuations, a significance threshold is introduced. A window is assigned a fault label only if the drift component within it exceeds this threshold.

(2) Short-Term Fault Sample Construction: To prevent the dilution or truncation of transient features during sliding window segmentation, a complete short-term fault is directly injected into a single signal segment of the “baseline analysis window length.”(Figure 7) This entire segment constitutes an independent sample, a method that preserves the integrity of the fault features while maintaining a uniform sample dimension.

(3) Compound Fault Sample Construction: To simulate complex fault superimposition, a long-term fault is first injected globally into the base signal, followed by the addition of a short-term fault at a random time point. Samples are generated using the same sliding window approach (Figure 8). To ensure labeling accuracy, a window is assigned a compound fault label only if two conditions are met simultaneously: first, the long-term fault component must exceed the aforementioned significance threshold; second, to address the issue of feature submergence when a short-term fault occupies only a small portion of a window, an overlap ratio threshold is introduced. A window is identified as a valid compound fault sample only if the duration of the short-term fault exceeds this overlap ratio, thereby preventing mislabeling due to minor overlaps.

All fault injection parameters were randomly drawn from predefined ranges to enhance sample diversity. Finally, Random Oversampling with Replacement was applied to balance the dataset by increasing the number of samples in minority fault classes, thereby improving the model’s generalization capabilities.

### 2.3. Specific Dataset Construction

Based on the differentiated sample construction method described above, this study utilized healthy sensor signals (sampling frequency 100 Hz) obtained from the full-scale Accelerated Pavement Testing (APT) facility at Changsha University of Science & Technology as the data foundation. To adequately capture the evolutionary characteristics of long-term faults, a 100-s (10,000-point) signal was selected as the injection base. The drift fault was initiated at 15 s. Through experimentation, the baseline window length was determined to be 500 points (5 s), with a step size of 25 points. The diagnostic threshold for drift and the overlap ratio threshold for short-term faults were set to 5% and 20%, respectively.

To implement the “Decomposition-Focus-Fusion” training strategy (detailed in Section 3.4), three distinct datasets were generated: a Short-Term Fault Dataset and a Long-Term Fault Dataset for the independent pre-training of the specialist sub-models, and a comprehensive Mixed-Fault Dataset for the subsequent training and evaluation of the final fusion model. The composition of these purpose-driven datasets is detailed in Table 2.

The class distribution in Table 2 reveals a notable imbalance, with the ‘drift’ class being the largest. This imbalance is a direct consequence of the differentiated sample construction method (Section 2.2). ‘Drift’ samples are generated by applying a sliding window to a long signal with a globally injected continuous fault—a process that naturally yields a large number of samples—whereas short-term faults are injected into discrete signal segments. To mitigate the risk of overfitting potentially introduced by this class imbalance and the use of oversampling, a comprehensive suite of regularization techniques was implemented during model construction and training. These include high-rate Dropout, an Early Stopping mechanism, a staged training and fine-tuning approach, Weight Decay (L2 regularization), and the use of a Focal Loss function.

## 3. Methodology: A Fault Diagnosis Framework Integrating Wavelet Analysis and Staged LSTM Training

### 3.1. Overall Framework

**Figure 9:**
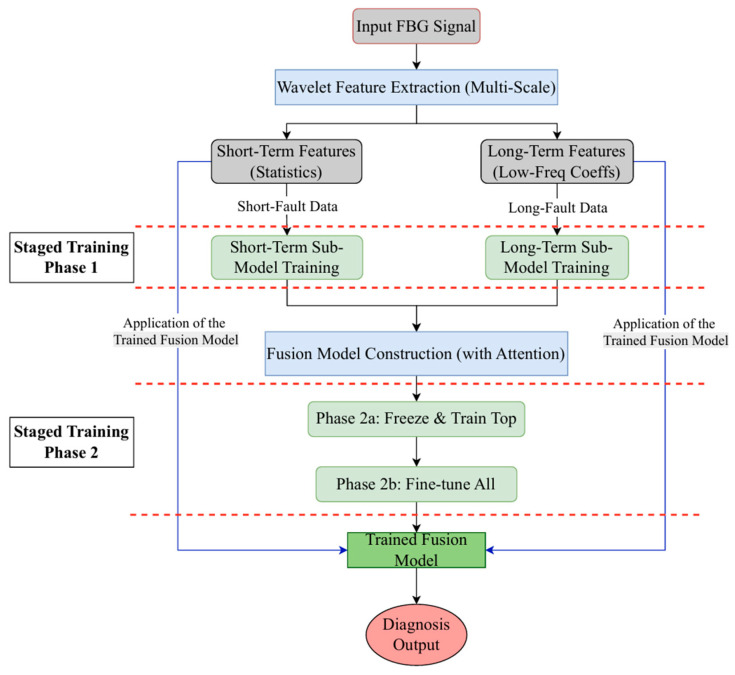
Overall Fault Diagnosis Framework Diagram.

To effectively diagnose the multi-mode faults described above, this study proposes an intelligent diagnostic framework based on a “Decomposition-Focus-Fusion” philosophy. This architecture is designed to circumvent the inherent learning interference and optimization difficulties that arise when processing heterogeneous features within a single, complex model. As illustrated in Figure 9, the framework is implemented in three stages: first, multi-scale features are extracted using the Discrete Wavelet Transform (DWT); subsequently, LSTM sub-models specializing in short-term and long-term faults are independently optimized through staged training; finally, a fusion network integrating an attention mechanism intelligently weights and combines the sub-model features, followed by global fine-tuning, to achieve precise classification of eight distinct sensor operational states.

### 3.2. Multi-Scale Feature Extraction via Wavelet Transform

Considering the non-stationary nature of sensor signals and their multi-scale fault characteristics, this study employs the Discrete Wavelet Transform (DWT) as the primary feature extraction tool. Leveraging its superior time-frequency localization capabilities, the DWT iteratively decomposes a signal into low-frequency approximation coefficients (A), which capture trend information, and high-frequency detail coefficients (D), which contain detail information. This process effectively reveals signal behaviors across different time scales. The Daubechies 4 (db4) wavelet was chosen as the basis for the transform. This selection is based on its excellent balance between smoothness and compact support. To minimize the boundary effects inherent in the DWT of finite-length signals, the ‘symmetric’ padding mode was employed for all wavelet decompositions. This standard method mitigates artifacts by creating a mirrored extension of the signal at its boundaries and is widely adopted as a best practice.

(6) $$A_{j}\left[k\right]=\sum_{n}h\left[n-2k\right]A_{j-1}\left[n\right]$$

(7) $$D_{j}\left[k\right]=\sum_{n}g\left[n-2k\right]A_{j-1}\left[n\right]$$

**Figure 10:**
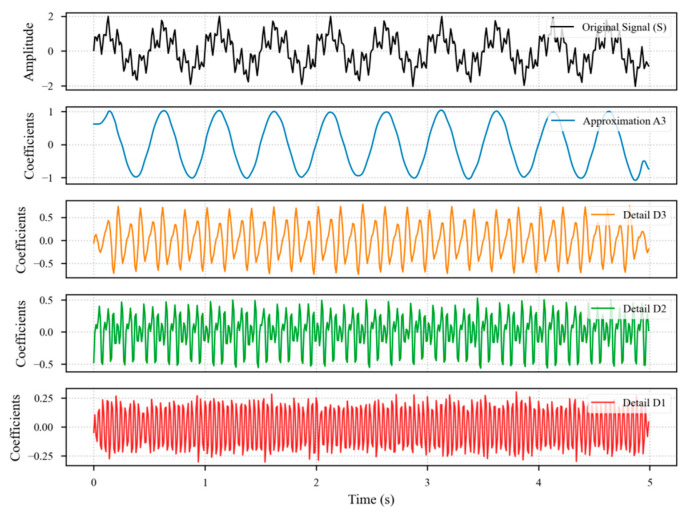
Discrete Wavelet Transform Multi-level Decomposition Schematic Diagram.

At decomposition level *j*, the signal *A*_*j*−__1_ is decomposed into approximation coefficients *A_j_* and detail coefficients *D_j_*, where *h*[*n*] and *g*[*n*] are the coefficients of the low-pass and high-pass filters, respectively, and *A*_0_ is the original signal (Figure 10). Based on the physical characteristics of different faults, a differentiated feature extraction scheme was adopted.

**Figure 11:**
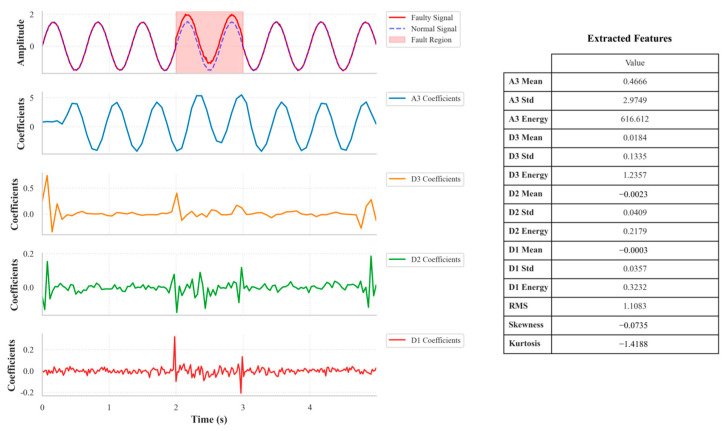
Schematic Diagram of Wavelet Decomposition and Short-term Feature Extraction.

Short-term faults manifest as local transients and energy anomalies in the signal, with features widely distributed across various frequency bands. Therefore, for all coefficients from a 6-level db4 wavelet decomposition (*A*_6_, *D*_1_–*D*_6_) and the original signal window itself, a comprehensive set of statistical features (i.e., mean, standard deviation, max, min, energy, mean absolute value, median, crest factor, RMS, skewness, and kurtosis) was extracted, the schematic illustration is presented in Figure 11. This creates a high-dimensional feature vector that comprehensively captures the instantaneous response across the entire frequency domain when a fault occurs.

Long-term drift faults, conversely, are fundamentally characterized by slow, trending changes in the signal’s baseline, which are primarily reflected in the low-frequency components. Consequently, only the lowest-frequency approximation coefficients (A6) were extracted as a feature sequence. This approach effectively isolates and focuses on the long-term evolutionary trend of the signal while filtering out high-frequency components that contribute little to identifying gradual changes and may even introduce interference.

Finally, to eliminate dimensional discrepancies and optimize model training, the two sets of extracted features were independently standardized using a StandardScaler. This transformer standardizes features by scaling them to a distribution with a mean of 0 and a standard deviation of 1, according to the formula:

(8) $$z=\frac{\left(x-\mu \right)}{\sigma }$$

where *x* is the original feature value, and *μ* and *σ* are the mean and standard deviation of that feature, respectively, calculated from the training set.

### 3.3. Modular Network Design

To effectively address the feature heterogeneity of short-term and long-term faults and to meticulously handle complex compound faults, this study adopted a modular network design. This approach first involves constructing “expert” sub-models, each specializing in a specific fault type. These are then integrated via a carefully designed fusion network to leverage their collective strengths, enabling precise discrimination of all defined fault states. All models are primarily constructed using Long Short-Term Memory (LSTM) units, whose ability to capture temporal dependencies is the cornerstone of our design.

#### 3.3.1. Working Principle of LSTM Unit

A Long Short-Term Memory (LSTM) network is a special type of Recurrent Neural Network (RNN) widely used for processing and predicting time-series data. Its key advantage lies in its sophisticated gating mechanism, which enables it to effectively learn and remember long-term dependencies in signals—a critical capability for capturing both the gradual trends and transient features of sensor data.

**Figure 12:**
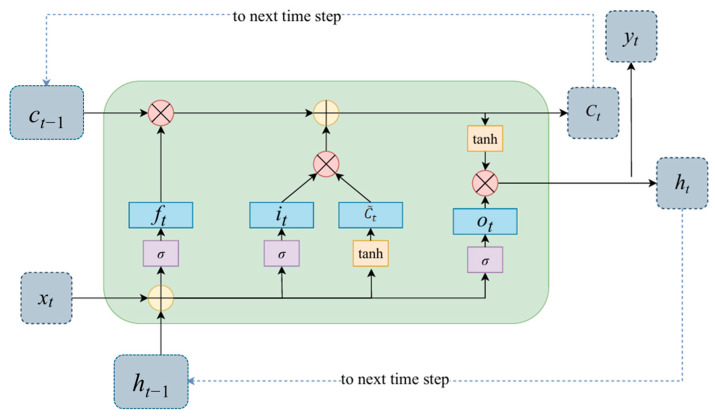
Schematic Diagram of an LSTM Unit.

Specifically, each LSTM unit (see Figure 12) dynamically regulates the flow of information through three crucial gates: an input gate (*i_t_*), a forget gate (*f_t_*), and an output gate (*o_t_*). These gates work in concert to selectively determine what historical information should be discarded, what new information should be stored in the memory cell, and what information should be output at the current time step. This intelligent “memory” and “forgetting” capability allows LSTMs to overcome the vanishing gradient problem common in traditional RNNs, thereby enabling the precise capture of key temporal patterns in complex, long-sequence data.

#### 3.3.2. Sub-Model Design and Fusion Model Construction

(1) Short-Term Fault Diagnosis Sub-Model

**Figure 13:**
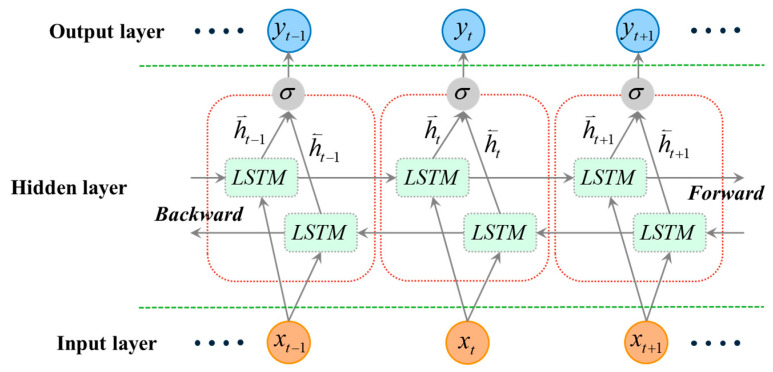
Schematic Diagram of Basic BiLSTM Structure.

**Figure 14:**
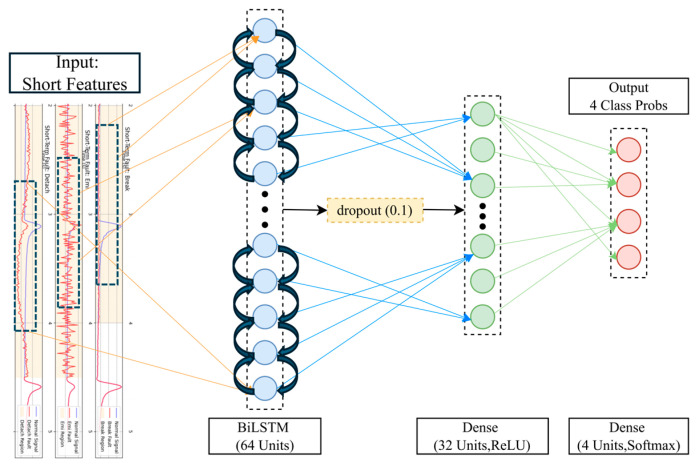
Architecture Diagram of Short-Term Fault Diagnosis Sub-Network.

This sub-model is designed to identify transient faults from the multi-scale wavelet features. Its core is a Bidirectional Long Short-Term Memory (Bi-LSTM) network, which captures deep dependencies among features. The Bi-LSTM layer is followed by a fully connected (Dense) layer for nonlinear feature integration and dimensionality reduction. Finally, a Softmax output layer maps the features to a probability distribution over four short-term states (“bias”, “gain”, “detachment”, and “normal (short-term)”). Dropout regularization is incorporated to enhance the model’s generalization capabilities. The detailed architecture is shown in Figure 13 and Figure 14.

(2) Long-Term Fault Diagnosis Sub-Model

**Figure 15:**
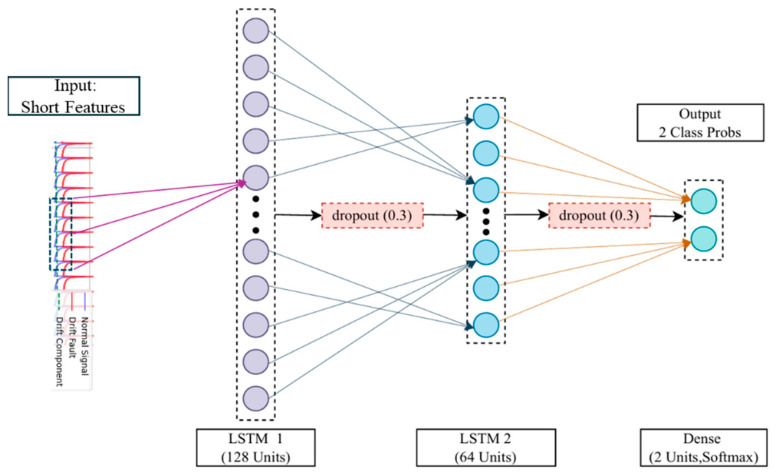
Architecture Diagram of Long-Term Fault Diagnosis Sub-Network.

This sub-model is dedicated to identifying slow-varying “drift” faults from the low-frequency wavelet approximation coefficients. To effectively capture its slowly evolving dynamics, the model employs a Stacked LSTM structure, consisting of two sequential LSTM layers, to learn deeper, more abstract temporal patterns. Similarly, Dropout regularization is applied to prevent overfitting. The model’s output layer uses a Softmax activation function to classify the sequence into one of two states: “normal” or “drift.” The detailed architecture is shown in Figure 15.

(3) Attention-Based Fusion Diagnosis Model

**Figure 16:**
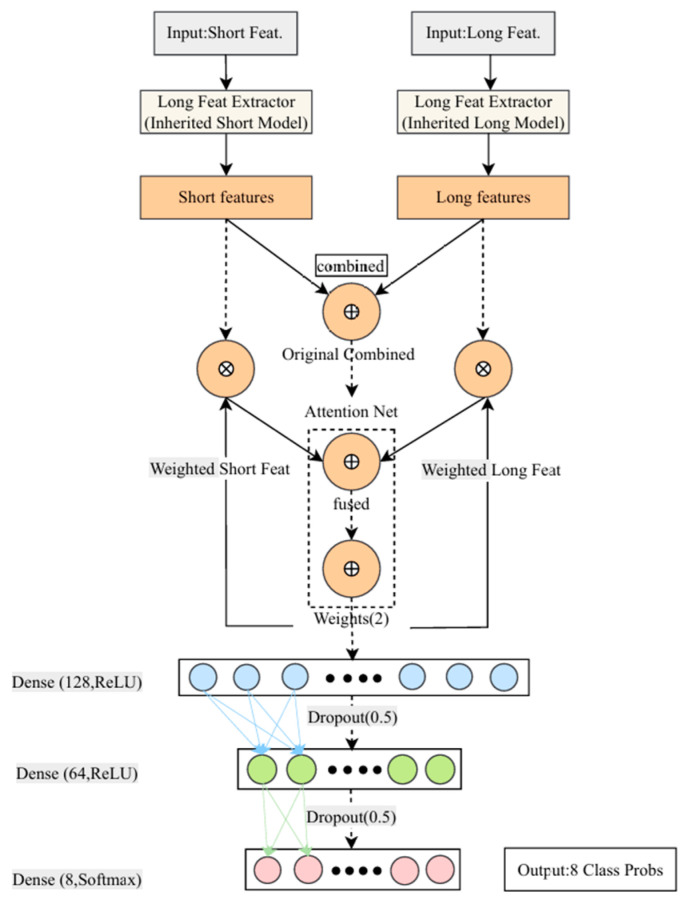
Detailed Architecture Diagram of Fusion Model.

The fusion model is the core of the entire framework, designed to intelligently integrate the feature extraction capabilities of the short-term and long-term sub-models. Its detailed architecture and data flow are illustrated in Figure 16, with the construction process as follows:

Feature Inheritance: The model first reuses the feature extraction backbones (i.e., all layers preceding the classification layer) from the two independently pre-trained sub-models. These are used to process the short-term statistical features and long-term low-frequency coefficients, respectively, and to output their learned high-level abstract features. This step provides the fusion model with a powerful initial feature extraction capability.

Dynamic Feature Weighting with an Attention Mechanism: The two high-level feature vectors are first concatenated and then fed into a specially designed fully connected attention network. This attention module dynamically learns and generates a set of attention weights based on the characteristics of the current input sample. These weights reflect the relative importance of the short-term and long-term feature streams to the final diagnosis in the current context.

Deep Fusion and Classification of Weighted Features: The learned attention weights are applied to their corresponding feature streams to achieve dynamic weighting. Subsequently, the weighted features are concatenated with the original (unweighted) features, forming a residual-like connection. This design allows the model to leverage the key information filtered by the attention mechanism while preserving the complete original context, thereby generating a more informative and intelligently regulated final feature representation. This final feature vector is then passed to a Classification Head, which consists of two sequential Dense and Dropout layers responsible for the final feature integration and nonlinear mapping. Finally, a Softmax output layer converts the processed feature vector into a probability prediction over the eight predefined fault states.

### 3.4. Staged Training Strategy

To effectively address the learning interference and optimization difficulties caused by feature heterogeneity when training a single complex model directly, this study employs a staged training strategy. The core idea of this strategy is “divide and conquer, then fine-tune.” First, the sub-models are allowed to “focus” on their respective “clean” datasets to become “experts” in their specific domains. Then, on this foundation, the entire network is integrated and fine-tuned to efficiently and robustly identify all fault modes, including compound faults. This process involves two main stages:

Stage One: Independent Sub-Model Pre-training. The two sub-models are trained independently on their respective short-term and long-term fault datasets, as constructed in Section 2.2. The purpose of this stage is to allow each sub-model to deeply mine and master the unique patterns of its corresponding fault type without interference from the other heterogeneous features. The specific training parameters are detailed in Table 3 and Table 4.

Stage Two: Fusion Model Training and Fine-tuning. This stage is crucial for achieving a precise final diagnosis of all eight states. It is conducted on the mixed dataset containing all fault types and is further divided into two steps: (1) Initial Fusion Training: The fusion model is constructed by loading the weights of the pre-trained sub-models from Stage One. These inherited feature extraction layers are frozen, and only the upper fusion and classification modules are trained. This allows the newly added attention network and classification head to learn how to integrate the features provided by the two “expert” sub-models without disrupting the well-trained underlying networks with large initial gradient updates. (2) Global Fine-tuning: All layers in the fusion model, including the underlying feature extractors, are unfrozen to allow all parameters to be updated. Training continues on the mixed fault dataset, but with a smaller learning rate, to perform end-to-end fine-tuning of the entire network. The training parameters for these two steps are provided in Table 5 and Table 6.

### 3.5. Implementation Details

To ensure the transparency and reproducibility of this study, the key hardware, software, and parameters used for implementation are summarized in Table 7. Detailed architectural and training hyperparameters for each model, such as network layer dimensions, learning rates, and batch sizes, are specified in their respective sections alongside the model diagrams (Figure 14, Figure 15 and Figure 16) and parameter tables (Table 3, Table 4, Table 5 and Table 6).

## 4. Experimental Setup, Results, and Discussion

### 4.1. Validation Case

To ensure the practical engineering relevance of this study, the baseline healthy data were sourced from the full-scale Accelerated Pavement Testing (APT) facility at Changsha University of Science & Technology. This facility, a large-scale indoor linear trafficking device, is a core technological means in pavement engineering for simulating the long-term service behavior of road structures, accelerating damage accumulation, and acquiring critical performance evolution data.

**Figure 17:**
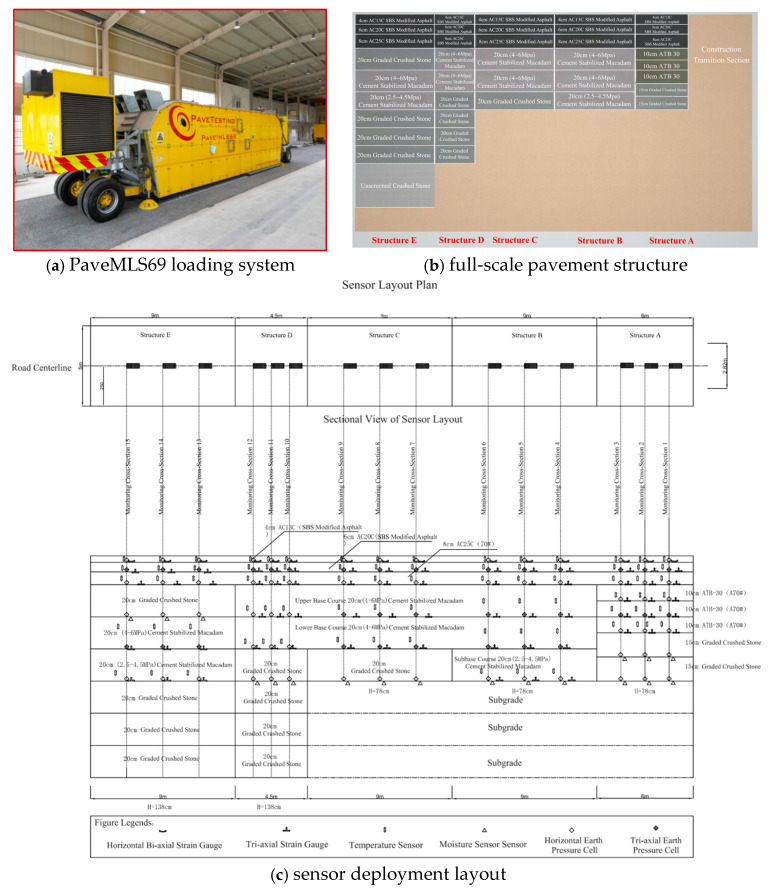
Full-scale test pavement and sensor deployment.

The APT facility features five representative asphalt pavement structures, including semi-rigid base, flexible base, full-depth, inverted, and a novel durable asphalt pavement structure (Figure 17b). The PaveMLS69 (Manufacturer: PaveTesting Limited, Letchworth, UK) loading system (Figure 17a) efficiently simulates heavy traffic loads, with key operational parameters—such as axle load (40–75 kN), tire pressure (500–1000 kPa), and loading speed—being precisely controllable. Data collected under this high-fidelity simulation of real-world service conditions formed the basis for the fault simulation and comprehensive dataset construction described in Section 2. This platform not only validates the effectiveness of the proposed method but also provides evidence for its potential application in real-world structural health monitoring [46].

### 4.2. Experimental Setup and Evaluation Metrics

(1) Comparative Methods

To comprehensively evaluate the performance of the proposed framework, three representative methods for benchmarking were selected: two classic machine learning models, namely Support Vector Machine (SVM) with a Radial Basis Function (RBF) kernel and K-Nearest Neighbors (KNN) with K = 5; and one end-to-end Unified LSTM-Model with a complexity comparable to that of our short-term sub-model. To ensure a fair comparison, all models were evaluated on the same comprehensive fault dataset constructed in Section 2.3. The input data were standardized: SVM and KNN used the same multi-scale wavelet features as our sub-models, while the Unified LSTM-Model received a unified input sequence formed by directly concatenating the short-term and long-term features.

(2) Evaluation Metrics

Performance was evaluated using the following core metrics: Overall Accuracy, Weighted F1-Score, Recall per Class, and the Confusion Matrix for error analysis. The detailed definitions of these metrics are provided in Table 8.

(3) Validation Strategy

Two validation strategies were employed to comprehensively assess model performance. The primary analysis in this study was conducted using a single, stratified 80/20 train-test split, where 80% of the data were used for training and 20% for testing. To further validate the robustness of these findings and ensure they were not dependent on this specific data partition, a subsequent 5-fold cross-validation was also performed. The results from the initial 80/20 split are presented for the main comparative analysis, while the cross-validation results, reported as mean ± standard deviation, are provided as a supplementary validation of the model’s stability and reliability.

### 4.3. Sub-Model Performance and Generalization Analysis

#### 4.3.1. Performance on the Standard Test Set

**Figure 18:**
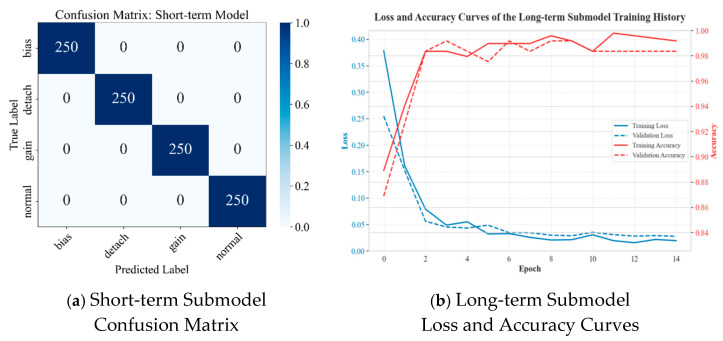
Performance of Submodels.

Before evaluating the final fusion model, the performance of the independently pre-trained short-term and long-term fault diagnosis sub-models was examined to confirm their effectiveness as foundational modules. The experimental results (Figure 18) show that the short-term fault sub-model performed perfectly on its specialized test set, achieving an accuracy and a weighted F1-score of 1.0000. Its confusion matrix was completely diagonal, demonstrating the high efficiency and accuracy of the extracted multi-scale wavelet statistical features and the Bi-LSTM architecture in capturing and distinguishing transient fault characteristics. Similarly, the long-term fault sub-model also excelled on its dedicated test set containing only “normal” and “drift” states, achieving a test accuracy of 0.9935. This confirms the success of the strategy that combines lowest-frequency wavelet approximation coefficients with a stacked LSTM network for identifying slow drift trends. The outstanding performance of these two sub-models validates that the “expert” modules in our modular design possess a powerful capability for single-fault-type identification, laying a solid foundation for effective subsequent feature fusion.

#### 4.3.2. Generalization and Robustness Analysis

Furthermore, the robustness of the sub-models against a reduced signal-to-noise ratio was evaluated. To simulate this condition, an additional layer of Gaussian white noise, with a standard deviation equivalent to 2.5% of each signal’s dynamic range, was added to the standard test set. The comparative performance of the sub-models under these conditions is summarized in Table 9.

The results in Table 9 indicate that, as expected, performance moderately decreased with the introduction of additional noise. The short-term model’s accuracy saw a degradation of approximately 3.5%, while the long-term model’s accuracy decreased by about 4.2%. Crucially, both models maintained a high accuracy of over 95%. This demonstrates a graceful degradation rather than a catastrophic failure, providing strong evidence that the learned features are robust and resilient to a realistic level of signal interference, thereby confirming the models’ potential for reliable field deployment.

### 4.4. Fusion Model Performance and Comparative Analysis

**Figure 19:**
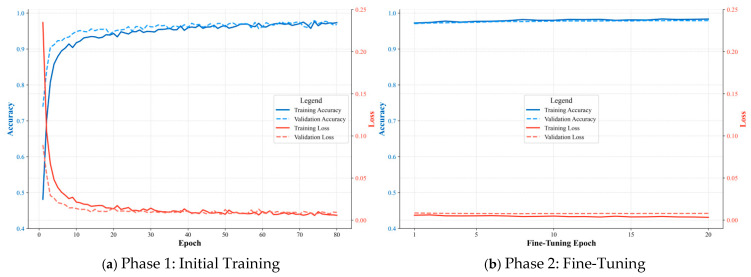
Training and validation history of the Fusion Model.

To validate the training process and confirm the absence of overfitting, the training and validation history of the Fusion Model is presented in Figure 19.

The plots demonstrate a healthy training dynamic across both phases. The training and validation curves for accuracy and loss track each other closely, without any significant divergence. This confirms the effectiveness of the anti-overfitting strategies, thereby validating the generalization capability of the final model evaluated below.

**Figure 20:**
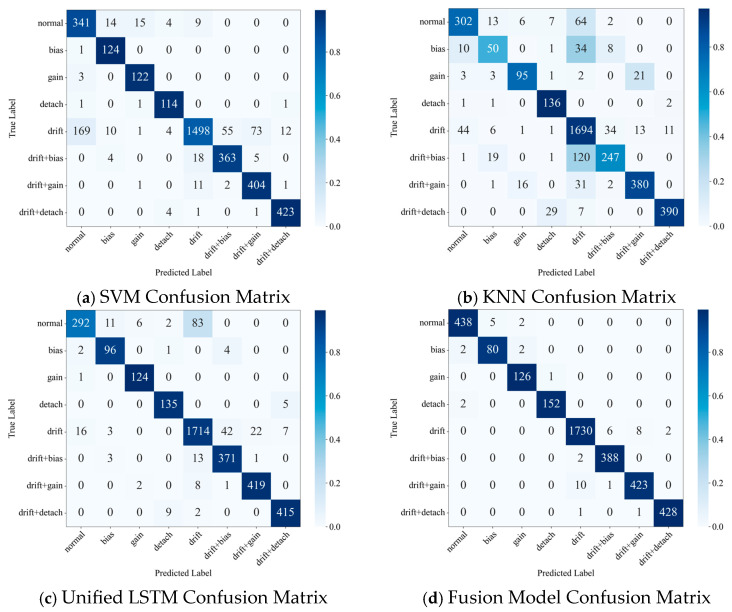
Confusion Matrices of Different Models on the Mixed Fault Test Set.

Figure 20 and Table 10 summarize the confusion matrices and overall performance metrics of all models on the entire test set, providing an initial impression for the subsequent in-depth analysis.

As can be seen from Table 10, the proposed Fusion Model significantly outperforms all comparative methods in both test accuracy and F1-score. To further reveal the deeper differences in the diagnostic capabilities of the models, the following sections will conduct a detailed comparative analysis of their performance from two key dimensions: single-fault and compound-fault diagnosis.

#### 4.4.1. Comparative Performance in Single Fault Diagnosis

**Figure 21:**
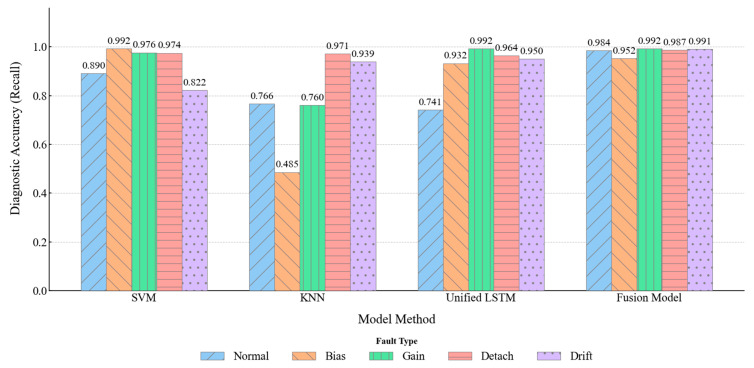
Model-Based Single Fault Diagnosis Comparison.

Accurate diagnosis of single faults, especially distinguishing between those with similar or subtle features, is critical for assessing a model’s foundational capabilities. Figure 21 illustrates the performance of each model on the five single-state classes.

As indicated by Figure 20 and Figure 21, distinguishing between the “normal” and “drift” states constitutes the primary challenge in single-fault diagnosis. The Fusion Model achieved a near-perfect distinction between “normal” (accuracy: 0.9843) and “drift” (accuracy: 0.9908) states, with virtually no mutual misclassification. This success is attributed to its long-term sub-model’s focused learning on the characteristic slow-varying trend of drift. In contrast, SVM and KNN, which rely on static feature snapshots and lack temporal modeling capabilities, exhibited severe confusion between these two states: SVM misclassified 169 “drift” samples as “normal,” while KNN misclassified 64 “normal” samples as “drift.” Although the Unified LSTM-Model possesses temporal capabilities, its unified architecture for processing heterogeneous features struggled to adequately focus on the key slow-varying characteristics that differentiate “normal” from “drift,” resulting in an accuracy of only 0.7411 for the “normal” state and significant mutual misclassification.

For the other three single-fault types with relatively distinct features, the Fusion Model also performed exceptionally well: “bias” (0.9524), “gain” (0.9921), and “detachment” (0.9870). This is credited to its short-term sub-model’s precise capture of sudden changes in statistical properties during transient faults. SVM performed well in identifying faults that cause distinct and stable changes in signal statistics, such as “bias” (0.9920) and “gain” (0.9760), but its accuracy slightly decreased for the more complex “detachment” fault (0.9421). KNN’s performance was notably poor on “bias” (0.4854) and “gain” (0.7600), as its distance-based metric is prone to sharp performance degradation when feature distributions are not compact or class boundaries are ambiguous; its accuracy on “detachment” (0.8144) was also suboptimal. The Unified LSTM-Model’s accuracy on these three short-term faults (0.9320, 0.9920, and 0.9375, respectively) was superior to that of SVM and KNN, demonstrating LSTM’s ability to learn temporal patterns. However, due to potential interference from the long-term feature information flow on the learning of short-term fault features within the unified model, its balance and overall precision still fell short of the Fusion Model.

#### 4.4.2. Comparison of Compound Fault Diagnosis Performance

**Figure 22:**
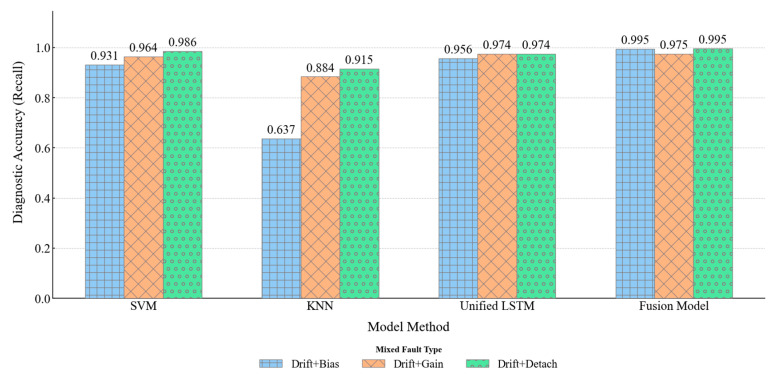
Model-Based Compound Fault Diagnosis Comparison.

The diagnosis of compound faults poses a higher demand on an algorithm’s feature decoupling capability. As revealed by the confusion matrices in Figure 20 and Figure 22, the core challenge lies in distinguishing between a pure “drift” fault and a “drift + X” type compound fault, where a transient disturbance is superimposed.

The baseline models exhibited clear deficiencies in this regard. Both SVM and KNN generated substantial bi-directional confusion between pure “drift” and compound faults (SVM misclassified 55 “drift” samples as compound, while KNN misclassified 120 compound samples as “drift”). The root cause is their inability to separate superimposed patterns from static feature snapshots. The Unified LSTM-Model, although an improvement, still showed significant feature confusion (42 “drift” samples were misclassified), indicating that the direct input of heterogeneous features in a single model induces learning interference, hindering effective decoupling of the background and the disturbance.

In stark contrast, the Fusion Model demonstrated exceptional diagnostic capability, achieving near-perfect identification of all three compound fault types (accuracy > 0.97 for all) with almost no confusion with single faults (only 6 misclassifications). This success is attributed to its staged learning, which provides clean, single-fault representations, and its attention mechanism, which can then intelligently identify the specific transient disturbances superimposed on the “drift” background.

Furthermore, an interesting “accuracy paradox” was observed in the experimental results: for both KNN and the Unified LSTM, the diagnostic accuracy for certain compound faults (e.g., “drift + bias”) was higher than that for their corresponding single faults (e.g., “bias”). This is likely not due to an improvement in model capability but rather a statistical artifact—the combination of a weak feature and a strong feature becomes more separable in the feature space. Interestingly, the Fusion Model also exhibited a similar trend (“drift + bias” at 0.9949 > “bias” at 0.9524), but this reflects a true synergistic enhancement effect. In this mechanism, the long-term sub-model’s precise capture of the “drift” background provides a strong context, enabling the attention mechanism to more robustly identify the superimposed weak transient disturbance, which further highlights the inherent advantages of its architecture.

#### 4.4.3. Robustness Validation with 5-Fold Cross-Validation

To provide a more robust evaluation of model performance and mitigate potential bias from a single data split, a 5-fold cross-validation was conducted on the entire mixed-fault dataset. For a fair comparison, all models were subjected to this validation procedure, with the final performance reported as the mean and standard deviation across the five folds. The overall performance comparison is presented in Table 11. To further dissect the diagnostic capabilities of each model, Table 12 provides a detailed comparison of the per-class recall (diagnostic accuracy) for all models across the eight distinct operational states.

The cross-validation results in Table 11 confirm the superior overall performance and stability of the proposed Fusion Model, as evidenced by its high mean accuracy and low standard deviation. Table 12 provides deeper insight, revealing that baseline models struggle significantly with specific, challenging classes. In contrast, the Fusion Model demonstrates consistently high recall and low variance across all fault types, including the complex compound faults, underscoring its reliability and advanced diagnostic capability.

#### 4.4.4. Out-of-Distribution Generalization Test

**Figure 23:**
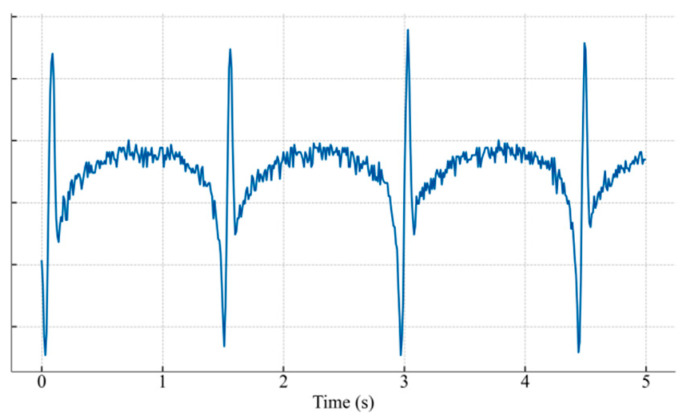
New Basis Signal.

**Figure 24:**
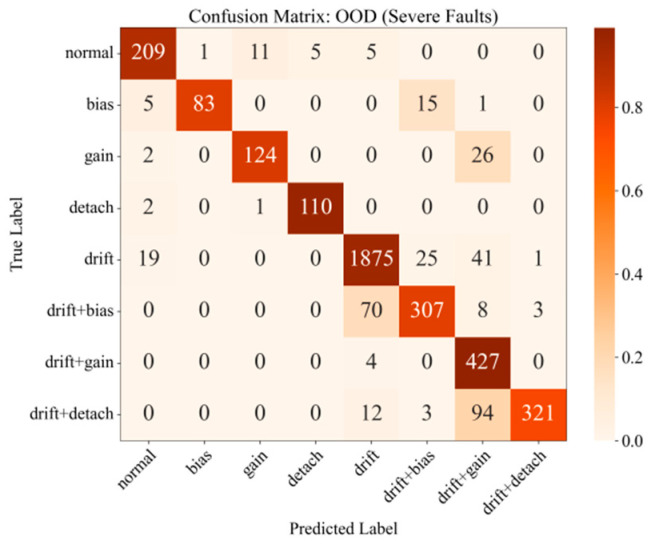
Confusion Matrix of the Fusion Model on the OOD Test Set.

To rigorously assess the generalization capability of the proposed framework, a stringent Out-of-Distribution (OOD) test was conducted, designed to simulate deployment in a completely novel engineering context. For this purpose, a new OOD test set was curated. The baseline healthy signals for this set originated from different road structures, sensor types, and axle loads, exhibiting distinct waveform characteristics compared to the training data (Figure 23). On this new baseline, faults of significantly greater severity than those seen during training were injected, with a parameter comparison detailed in Table 13. The model’s performance on this OOD test set is summarized in Table 14 and Figure 24.

As shown in Table 14, the model’s accuracy exhibited a graceful degradation from 98.82% on the in-distribution test set to 90.71% when faced with the dual OOD challenges of unseen signal morphologies and severe faults. This result strongly indicates that the fundamental, generalizable physical signatures of faults were learned, rather than the model merely overfitting to the training data distribution. Consequently, robust diagnostic performance is maintained even in a more challenging and previously unseen environment.

The confusion matrix (Figure 24) provides deeper insights into the model’s behavior under extreme conditions. The primary misclassification, for instance, was the confusion of drift + detach with drift + gain. This suggests that on the new signal baseline, the feature representation of a severe detachment fault superimposed on a drift trend became highly similar to that of a severe gain fault under the same drift condition. This finding not only highlights the inherent complexities of real-world diagnostics but also underscores the framework’s sensitivity in capturing subtle feature variations.

### 4.5. Ablation Study

To systematically deconstruct the framework and quantify the contribution of its key architectural and methodological components, a series of ablation experiments were conducted. The performance of the full model was compared against several degraded versions, with the results summarized in Table 15.

The ablation results in Table 15 reveal a clear synergistic effect between the framework’s components. The transition from raw data (85.17%) to multi-scale DWT features (93.60%) establishes the critical role of feature extraction as the foundation for high performance. Building on this, the learning strategy proves paramount; the poor performance of the end-to-end trained fusion model (91.76%) confirms that the staged ‘Decomposition-Focus-Fusion’ paradigm is essential to resolve optimization conflicts between the heterogeneous feature streams. Finally, the attention mechanism provides the decisive refinement, with its inclusion (98.82%) yielding a significant boost over simple feature concatenation (93.99%) by intelligently resolving feature conflicts during fusion. Therefore, the framework’s success relies on this synergy: DWT provides discriminative features, staged training enables their effective learning, and attention optimizes their final fusion.

### 4.6. Analysis of the Attention Mechanism

**Figure 25:**
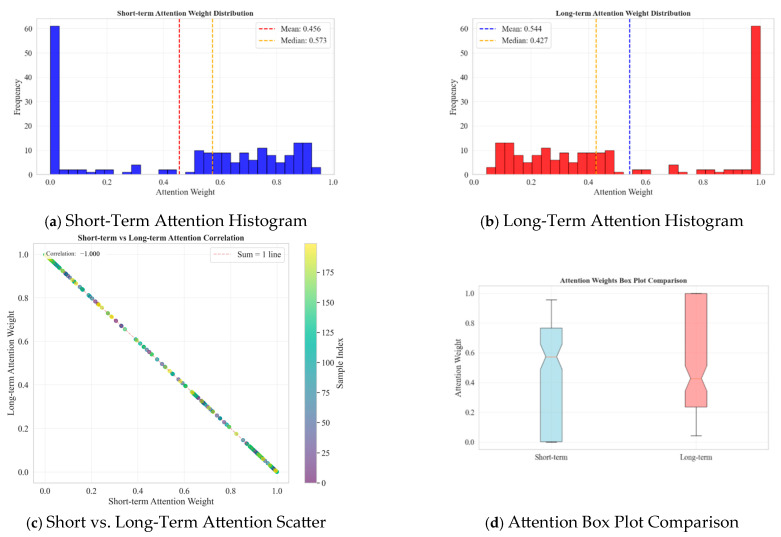
Distribution of attention weights for extracted samples.

To further investigate the role of the attention mechanism in feature fusion, the attention weight distributions corresponding to randomly selected samples from the test set are visualized in Figure 25a–d.

As shown in Figure 25a,b, the attention weights for the short-term and long-term features exhibit distinct peaks near 0 and 1, respectively (with means of 0.456 and 0.544), indicating that the model does not statically or uniformly allocate weights. Instead, when a feature of one time scale (e.g., “drift”) dominates, its weight approaches 1, while the weight of the other feature correspondingly approaches 0. This dynamic trade-off is further confirmed by the scatter plot in Figure 25c, where the weight data points are tightly clustered around the line of sum-to-one (with a Pearson correlation coefficient close to −1.0). The box plot in Figure 25d compares the statistical distributions of the two weights, showing that the long-term feature weights have a greater dispersion and more outliers in the high-weight region. This may reflect the model’s heightened focus on diverse and persistent “drift” patterns. These visualizations collectively confirm that the attention mechanism can intelligently assign weights to features of different time scales based on the real-time characteristics of the input signal, achieving effective fusion of heterogeneous information. This is a key technical support for the exceptional diagnostic performance of the Fusion Model. Additionally, the introduction of Focal Loss, by compelling the model to focus on hard-to-classify samples, further enhanced its ability to discriminate subtle fault differences and effectively addressed the data imbalance problem.

### 4.7. Real-Time Diagnostic Potential Assessment of the Fusion Model

To evaluate the online diagnostic efficacy of the Fusion Model in practical engineering applications, a simulated continuous data stream fault diagnosis experiment was designed and conducted. This experiment used a 100-s healthy sensor signal as a base, onto which various single and compound faults were dynamically introduced at different time points according to the pre-defined fault injection strategy, thereby simulating a real-world fault evolution process. Subsequently, the continuous fault signal stream was segmented using a sliding window (length: 500, step: 25) and fed into the trained Fusion Model for real-time diagnosis.

**Figure 26:**
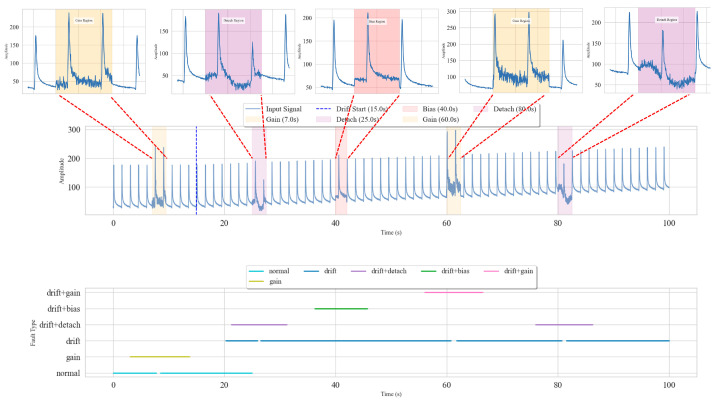
Simulation results of real-time diagnosis.

As shown in Figure 26, the Fusion Model accurately tracked and identified the various fault types as they dynamically appeared in the continuous signal stream. The model not only successfully distinguished between transient faults like “bias,” “gain,” and “detachment” and accurately identified the long-term “drift” fault, but it also demonstrated high-precision discrimination of complex compound faults (e.g., “drift + bias”). The diagnostic results highly matched the injection times and types of the true faults, proving the model’s fast response capability to dynamic fault evolution. A noteworthy detail is that in the 15–20 s interval, the model did not immediately detect the injected “drift” fault because its signal variation was extremely subtle in the initial stage. However, once the feature became more pronounced, the model quickly and accurately identified it, which aligns with the logic of progressive identification of weak faults in practical applications.

**Figure 27:**
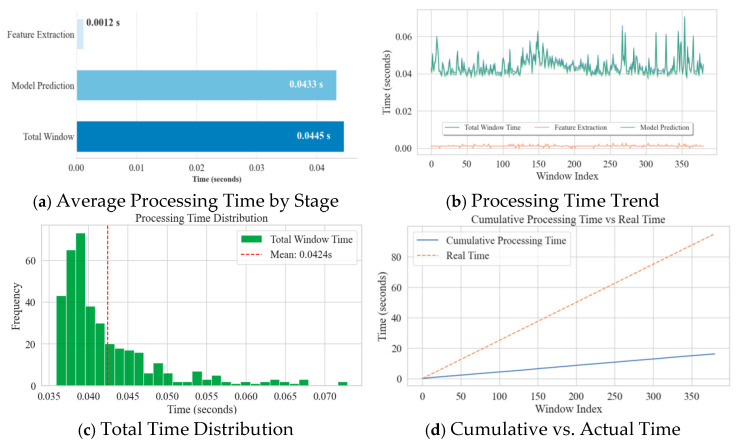
Statistics of real-time diagnosis processing time for the Fusion Model.

To quantitatively assess the model’s computational efficiency, the processing time at various diagnostic stages was further analyzed, as shown in Figure 27a–d.

The analysis of average time consumption per stage (Figure 27a) shows that although model prediction was the main computational overhead (0.0433 s), efficient feature extraction (0.0012 s) kept the total average processing time per window to approximately 0.0445 s. The processing time trend (Figure 27b) and distribution histogram (Figure 27c) further confirmed the high stability and consistency of the model’s processing speed, with no significant abnormal fluctuations. Most critically, the comparison of cumulative processing time versus actual time (Figure 27d) clearly shows that the total time to process the entire 100-s signal stream (381 windows) was only 16.9 s. This means that the model’s average processing speed (0.044 s/window) was far faster than the data generation speed (0.25 s/window), with a real-time processing ratio much greater than 1.

In summary, the continuous data stream diagnosis experiment and processing time statistical analysis collectively validate that the proposed fusion diagnostic framework not only exhibits high accuracy and rapid response to dynamic fault evolutions but also possesses excellent computational efficiency, meeting the requirements for real-time online monitoring. This demonstrates its application potential in condition monitoring and fault warning for embedded sensors in road engineering.

### 4.8. Discussion

The proposed supervised framework is subject to two limitations inherent to its data-driven nature: its reliance on a synthetic dataset, which may not fully capture the variability of real-world failures, and its dependence on a predefined set of fault modes, which can be challenged by data scarcity or novel faults in the field. However, the primary contribution and generalization potential of this study lie in its “Decomposition-Focus-Fusion” architecture, which is designed as a modular and extensible blueprint to address these long-term challenges. Its adaptability allows practitioners to integrate new sub-models for novel fault types and retrain the attention layer to learn new feature relationships. Furthermore, for highly complex diagnostic scenarios involving numerous sub-models, the current fusion layer could be enhanced by adopting more advanced attention mechanisms, such as multi-head or self-attention, to capture intricate inter-dependencies between the various fault feature streams. This makes the core methodology a scalable and generalizable paradigm for complex sensor fault diagnosis tasks.

## 5. Conclusions

To address the diagnostic challenges posed by embedded sensors in asphalt pavement under complex service conditions, this study proposed and validated an intelligent diagnostic framework that integrates a Discrete Wavelet Transform (DWT) with a staged, attention-based LSTM network. The main conclusions are as follows:

(1) The proposed differentiated sample construction and fine-grained labeling strategy effectively address the challenge of data scarcity and labeling ambiguity. By employing targeted injection, segmentation, and threshold control, this strategy resolves the inherent conflict in effectively representing long-term and short-term faults within a unified dimension, which arises from their conflicting time scales. This approach successfully yielded a high-quality dataset for model training and testing.
(2) The novel “Decomposition-Focus-Fusion” architecture successfully resolves the learning interference issue caused by heterogeneous features. Its superior performance was validated through rigorous 5-fold cross-validation, achieving a mean accuracy of 98.89%. Furthermore, a comprehensive ablation study empirically confirmed that each component of the proposed architecture is indispensable for this high performance, while dedicated generalization tests verified the robustness of the foundational sub-models. This evidence collectively demonstrates that the framework fundamentally overcomes the two core challenges: the confusion between “normal” and “drift” states and the decoupling of compound faults, performing significantly better than baseline models.
(3) The framework possesses strong potential for online, real-time diagnosis in engineering applications. Its processing efficiency, with an average time of just 0.0445 s per data window, far exceeds the data generation rate (0.25 s per window), and it accurately tracks dynamic faults. This confirms its high feasibility for practical deployment as an efficient and robust solution that ensures the integrity of monitoring data.

Although this study has achieved promising results, future work will focus on further enriching and refining the fault mode library and enhancing the model’s generalization capabilities based on more field-measured data.

## 6. Future Work

Building upon the findings and the limitations discussed, future research will proceed along three primary directions to bridge the gap from simulation to field application:

(1) High-Fidelity Data Acquisition. Controlled physical experiments, such as rutting plate tests with embedded sensors, will be conducted to generate high-fidelity fault data under realistic mechanical and thermal stresses for model validation and refinement.
(2) Advanced Methodologies for Data Scarcity. Methodologies such as transfer learning and meta-learning will be explored to enable the model to generalize from a limited number of labeled examples (“few-shot learning”).
(3) Architectural Enhancement of the Fusion Module. The current attention mechanism will be explored and potentially replaced with more advanced variants, such as multi-head attention or self-attention (as used in Transformers), to improve the framework’s capacity to fuse features from a larger and more complex set of expert sub-models in future iterations.

## Figures and Tables

**Table 1:** Fault Parameter and Value Range.

Fault Type	Parameters	Value Range
Bias	w	5~20% of the signal range
	$\sigma_{b}\left(t\right)$	Standard deviation is 1% of the signal range
Gain	k	1.2~1.5
	$\sigma_{g}\left(t\right)$	Standard deviation is 5% of the signal range
Detachment	$e^{-\alpha \cdot f\left(t^{\prime }\right)}$	Control signal decays to 40~60% of the original
	$d\left(t^{\prime }\right)$	A 0.5 Hz sinusoidal drift with 6% signal range
	$\sigma_{d}\left(t^{\prime }\right)$	Standard deviation is 5% of the signal range
Drift	$K_{1}$	0.002~0.005
	$K_{2}$	1 × 10^−5^~4 × 10^−5^

**Table 2:** Label Distribution of the Fault Dataset.

Dataset Type	Fault Type	Number of Samples
Short-Term Fault Dataset	normal	1250
	bias	1250
	gain	1250
	detach	1250
Long-Term Fault Dataset	normal	500
	drift	1024
Mixed Fault Dataset	normal	1777
	bias	500
	gain	509
	detach	617
	drift	6984
	drift + bias	1562
	drift + gain	1733
	drift + detach	1721

**Table 3:** Short-term Sub-model Training Parameters.

Parameter Category	Specific Content
Optimizer	AdamW, learning rate 0.0005, Weight Decay 1 × 10^−4^
Loss function	Sparse categorical crossentropy
Callback function	Early Stopping: patience value 20, restore best weights
Training configuration	Training epochs 100, batch size 64

**Table 4:** Long-term Sub-model Training Parameters.

Parameter Category	Specific Content
Optimizer	Adam(default parameters)
Loss function	Sparse categorical crossentropy
Class weight	Balanced class weights (used to handle class imbalance)
Training configuration	Training epochs 15, batch size 32

**Table 5:** Fused Model Initial Stage Training Parameters.

Parameter Category	Specific Content
Optimizer	Adam (default parameters)
Loss function	Focal Loss (gamma 2.0, alpha 0.25)
Class weight	Balanced class weights (used to handle class imbalance)
Training configuration	Training epochs 100, batch size 32

**Table 6:** Fused Model Overall Fine-tuning Stage Training Parameters.

Parameter Category	Specific Content
Optimizer	Adam, learning rate 1 × 10^−5^
Loss function	Focal Loss (gamma 2.0, alpha 0.25)
Class weight	Balanced class weights (used to handle class imbalance)
Callback function	Early Stopping: patience value 5, restore best weights
Training configuration	Training epochs 20, batch size 32

**Table 7:** Implementation Environment.

Category		Specification
Hardware Environment	Operating System	Microsoft Windows 11 Home (64-bit)
	Processor	13th Gen Intel(R) Core(TM) i9-13980HX
	Graphics Card	NVIDIA GeForce RTX 4060 Laptop GPU
Software Framework	Primary Language	Python 3.12
	Core Libraries	TensorFlow (2.18.0), Scikit-learn (1.5.2)
Reproducibility	Random Seed	A global seed of 42 was set for all stochastic processes in NumPy (2.0.2) and TensorFlow.

**Table 8:** Calculation Formulas for Evaluation Metrics.

Evaluation Metric	Calculation Formula	Parameter Explanation
Overall Accuracy	Accuracy = $\frac{N_{correct}}{N_{total}}$	*N_correct_*: Number of correctly predicted samples; *N_total_*: Total number of samples
Precision	Precision*_c_*$\;=\;\frac{TP_{c}}{TP_{c}+FP_{c}}$	*TP_c_*: Number of true positives for class c; *FP_c_*: Number of false positives for class c
Recall	Recall*_c_*$\;=\;\frac{TP_{c}}{TP_{c}+FN_{c}}$	$FN_{c}$: Number of false negatives for class c
F1 Score	*F* $1_{c}=2\frac{Precision_{c}\times Recall_{c}}{Precision_{c}+Recall_{c}}$	Precision*_c_*: Precision for class c; Recall*_c_*: Recall for class c
Weighted F1 Score	$\mathrm{Weighted}\;F1\;=\;\sum (\frac{N_{c}}{N_{total}}\times$ *F*1*_c_*)	$N_{c}$: Number of samples in class c; *F*1*_c_*: F1 Score for class c
Confusion Matrix	A *C* × *C* matrix	The element in the i-th row and j-th column is the number of samples actually in class i but predicted as class j

**Table 9:** Sub-Model Performance Under Increased Noise Conditions.

Sub-Model	Test Condition	Test Accuracy	F1 Score
Short-Term	Original Clean Test Set	1.0000	1.0000
	Noisy Test Set (+2.5% Noise)	0.9630	0.9628
Long-Term	Original Clean Test Set	0.9935	0.9935
	Noisy Test Set (+2.5% Noise)	0.9518	0.9513

**Table 10:** Performance Comparison of Different Models on the Mixed Fault Test Set.

Model	Test Accuracy	F1 Score
SVM	0.8895	0.8915
KNN	0.8646	0.8616
Unified LSTM-Model	0.936	0.9348
Fusion Model	0.9882	0.9882

**Table 11:** Overall Performance Comparison using 5-Fold Cross-Validation.

Model	Test Accuracy (Mean ± Std)	F1 Score (Mean ± Std)
SVM	0.9084 ± 0.0040	0.9083 ± 0.0038
KNN	0.8529 ± 0.0045	0.8486 ± 0.0051
Unified LSTM-Model	0.9420 ± 0.0057	0.9418 ± 0.0061
Fusion Model	0.9889 ± 0.0040	0.9889 ± 0.0040

**Table 12:** Per-Class Recall (Mean ± Std) Comparison using 5-Fold Cross-Validation.

Fault Type	SVM	KNN	Unified LSTM-Model	Fusion Model
normal	0.844 ± 0.007	0.776 ± 0.027	0.841 ± 0.056	0.9480 ± 0.0268
bias	0.754 ± 0.042	0.342 ± 0.070	0.903 ± 0.033	0.9858 ± 0.0079
gain	0.963 ± 0.020	0.744 ± 0.029	0.953 ± 0.015	0.9958 ± 0.0056
detach	0.959 ± 0.013	0.920 ± 0.018	0.979 ± 0.013	0.9899 ± 0.0075
drift	0.913 ± 0.005	0.927 ± 0.008	0.947 ± 0.009	0.9941 ± 0.0030
Drift + bias	0.862 ± 0.018	0.669 ± 0.021	0.963 ± 0.010	0.9815 ± 0.0041
Drift + gain	0.950 ± 0.010	0.883 ± 0.016	0.969 ± 0.008	0.9923 ± 0.0048
Drift + detach	0.970 ± 0.014	0.921 ± 0.009	0.981 ± 0.006	0.9901 ± 0.0049

**Table 13:** Fault Parameter Comparison.

Fault Type	Parameters	In-Distribution (Standard)	OOD (Severe)
Bias	w	[0.05, 0.20]	[0.24, 0.34]
	$\sigma_{b}\left(t\right)$	(0, 0.01) × R	(0, 0.012) × R
Gain	k	[1.2, 1.5]	[1.5, 1.9]
	$\sigma_{g}\left(t\right)$	(0, 0.05) × R	(0, 0.06) × R
Detachment	$e^{-\alpha \cdot f\left(t^{\prime }\right)}$	[0.4, 0.6]	0.75
	$d\left(t^{\prime }\right)$	0.06 × R	0.07 × R
Drift	*K*_1_	[0.002, 0.005]	[0.006, 0.010]

Note: R denotes the signal’s dynamic range (signal_range).

**Table 14:** Performance Comparison of the Fusion Model.

Test Condition	Test Accuracy	F1-Score
In-Distribution Test Set (80/20 Split)	0.9882	0.9882
Out-of-Distribution (OOD) Test Set	0.9071	0.9074

**Table 15:** Results of the Ablation Study.

Configuration	Test Accuracy	F1 Score
LSTM on Raw Data	0.8517	0.8448
Unified LSTM on DWT	0.9360	0.9348
Fusion Model w/o Staged Training	0.9176	0.9199
Fusion Model w/o Attention	0.9399	0.9414
Proposed Full Model	0.9882	0.9882

## Data Availability

The original contributions presented in this study are included in the article. Further inquiries can be directed to the corresponding authors.
